# Posttraumatic Stress Disorder Treatment Decision Aid: User-Centered Design Update Approach

**DOI:** 10.2196/89074

**Published:** 2026-06-10

**Authors:** Sadie E Larsen, Cybele Merrick, Yaara Zisman-Ilani, Karen B Eden, Shanna Treworgy, Jessica L Hamblen

**Affiliations:** 1 National Center for Posttraumatic Stress Disorder White River Junction, VT United States; 2 Department of Psychiatry and Behavioral Medicine Medical College of Wisconsin Milwaukee, WI United States; 3 Temple University Philadelphia, PA United States; 4 University College London London, England United Kingdom; 5 Oregon Health & Science University Portland, OR United States; 6 White River Junction VA Medical Center White River Junction, VT United States; 7 Geisel School of Medicine at Dartmouth Hanover, NH United States

**Keywords:** posttraumatic stress disorder, PTSD, shared decision-making, patient decision aids, treatment

## Abstract

**Background:**

Posttraumatic stress disorder (PTSD) is associated with significant health and societal economic burdens, making treatment a priority. There are effective treatments, but there is no indication at this point which treatments will work best for which patients. Patient decision aids (DAs) are evidence-based tools designed to support patients and providers in weighing the benefits and risks of different treatment options and in making value-informed choices as one part of shared decision-making. The National Center for PTSD published an online PTSD Treatment DA in 2017.

**Objective:**

This paper outlines a collaborative process for significantly updating the PTSD Treatment DA in line with newer clinical practice guidelines and updated technological needs.

**Methods:**

The development and updating process was conducted by a small team in consultation with an expert panel and consumer input. User experience testing with people who screened positive for PTSD focused on the experience of completing specific core tasks such as comparing treatment options using the DA. Field testing included Veterans Administration patients and providers using the DA in clinical appointments to gather feedback about feasibility, satisfaction, acceptability, balance, fidelity, decisional conflict, and decisional self-efficacy.

**Results:**

Pilot field testing suggested that the PTSD Treatment DA was acceptable, satisfactory, balanced, feasible to implement, and associated with improvements in decisional conflict and in decisional self-efficacy in this small sample. Design decisions based on stakeholder feedback are described, including which treatments to include, what design format to use, how to assess preferences, and how to most effectively convey treatment effectiveness information.

**Conclusions:**

The revised PTSD Treatment Decision Aid is freely available online and is consistent with the International Patient Decision Aid Standards.

## Introduction

Posttraumatic stress disorder (PTSD) is associated with significant individual health and societal economic burdens [1], making effective treatment a priority both within the Veterans Administration (VA) and in the general population. Importantly, there are treatments that work well, but there is no indication at this point which treatments will work best for which patients [2]. Shared decision-making (SDM) is a patient-centered health communication approach that helps patients make value- and preference-based treatment decisions that are informed by patient goals, provider recommendations, and best available evidence [3]. Ultimately, SDM helps involve patients in the decision about which treatment they prefer, thus respecting patient autonomy in the context of health care choices with no one right answer [4]. Greater patient involvement may also lead to better outcomes by enhancing engagement, increasing knowledge about treatment options, and accounting for patients’ needs, goals, and preferences, though empirical evidence is still nascent [4,5].

Patient decision aids (DAs) are evidence-based tools designed to support patients and providers (and sometimes family members) in weighing the benefits and risks of different treatment and service options as one part of the SDM process. A recent large systematic review and meta-analysis of DAs for all health conditions found that they helped to increase patient knowledge, accurate risk perceptions, and correspondence between patient values and options chosen while decreasing indecision about personal values, decisional conflict, and passivity in decision-making [6].

In 2017, the National Center for PTSD (NCPTSD) produced an online PTSD Treatment DA, guided by best practices as outlined by the International Patient Decision Aid Standards (IPDAS) Collaboration [7], highlighting the first-line treatments in the Veterans Affairs/Department of Defense (VA/DoD, 2017) Clinical Practice Guideline (CPG) for the management of PTSD [8]. In 2023, an updated VA/DoD CPG was published, prompting a need to revise the DA. In addition, a need was identified to update the technological capacity of the DA with a mobile-responsive design. Thus, in 2022, the NCPTSD started the process of revising the PTSD Treatment DA (we will here forward call the original the DA 1.0, and the newly published the DA 2.0).

**Figure 1:**
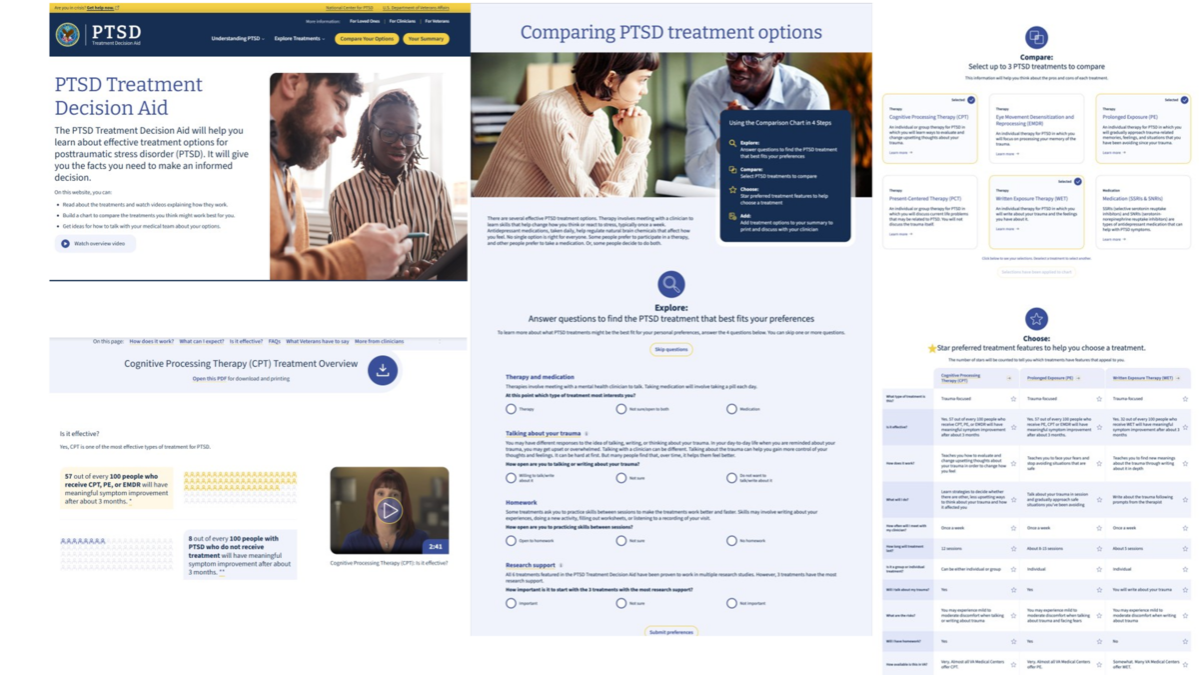
Images from the PTSD Treatment Decision Aid website. Selected pages from the PTSD Treatment Decision Aid show the homepage with top navigation bar, a snippet from a detailed treatment page showing a clinician video and a treatment effectiveness infographic, and 2 screenshots from the “compare your options” page where users can identify their preferences and compare options.

The PTSD Treatment DA 2.0 website (freely available online) [9] enables veterans or civilians with PTSD to learn about and compare effective treatments. It is designed to be used to support an SDM conversation, either on its own or with a clinician in an appointment. Six in-depth web pages highlight each main treatment (see “Treatments to Include” section) including videos of providers describing the treatments and infographics describing their effectiveness. A treatment comparison web page helps people clarify preferences (eg, “How open are you to talking or writing about your trauma?”) and compare the benefits and risks of the treatment options in a customizable chart. The DA 2.0 also includes general information about PTSD (including a PTSD self-screen) and PTSD treatment as well as frequently asked questions, a list of treatments in addition to the 6 highlighted in the DA, and how to talk to a clinician about PTSD treatment. As patients work through the DA, information is stored on “Your Summary,” a web page that can be printed to share with a clinician. There are also specific pages with information for veterans, loved ones, and clinicians. The overall goal of the DA 2.0 is to educate those with PTSD and empower them to explore preference- and value-informed treatment options so they can have a better SDM discussion with clinicians about choosing a PTSD treatment. Selected web pages from the website are shown in Figure 1.

This paper outlines the process used to update the existing PTSD Treatment DA. The aim was to use principles of adult learning, best practices in DA development, and user-centered web design and digital communication principles to update and produce a new PTSD Treatment DA 2.0.

## Methods

### Ethical Considerations

The proposed project (DA development, user experience testing, and field testing) was submitted to and reviewed by the Veteran’s Institutional Review Board (IRB) of Northern New England and determined not to be research but instead a quality improvement project that does not require IRB approval. The White River Junction VA Quality, Safety, and Value team also reviewed the project. The White River Junction VA Privacy Officer was consulted for proper handling of private information. For this portion of the project, all patients and participants were informed about the purpose of the development of the DA and were explicitly told that their choice to provide feedback was voluntary. They were asked not to provide identifying information to protect their privacy. The Veteran’s IRB of Northern New England also reviewed the needs assessment survey administered before the development of the DA 2.0, which was determined to be “not human subjects research” and therefore overseen by the Research and Development Committee (case number: 1702116-1). The IRB made this determination because the researchers did not have access to any Health Insurance Portability and Accountability Act (HIPAA) identifiers from the survey respondents. The company that collected the survey responses ensured that all identifiers were removed prior to sending the dataset to the authors. The company has very strict standards for data security and complies with all good clinical practice requirements. The data were submitted securely, and then stored on a VA-encrypted drive.

### Development Process and Team

#### Overview

**Figure 2:**
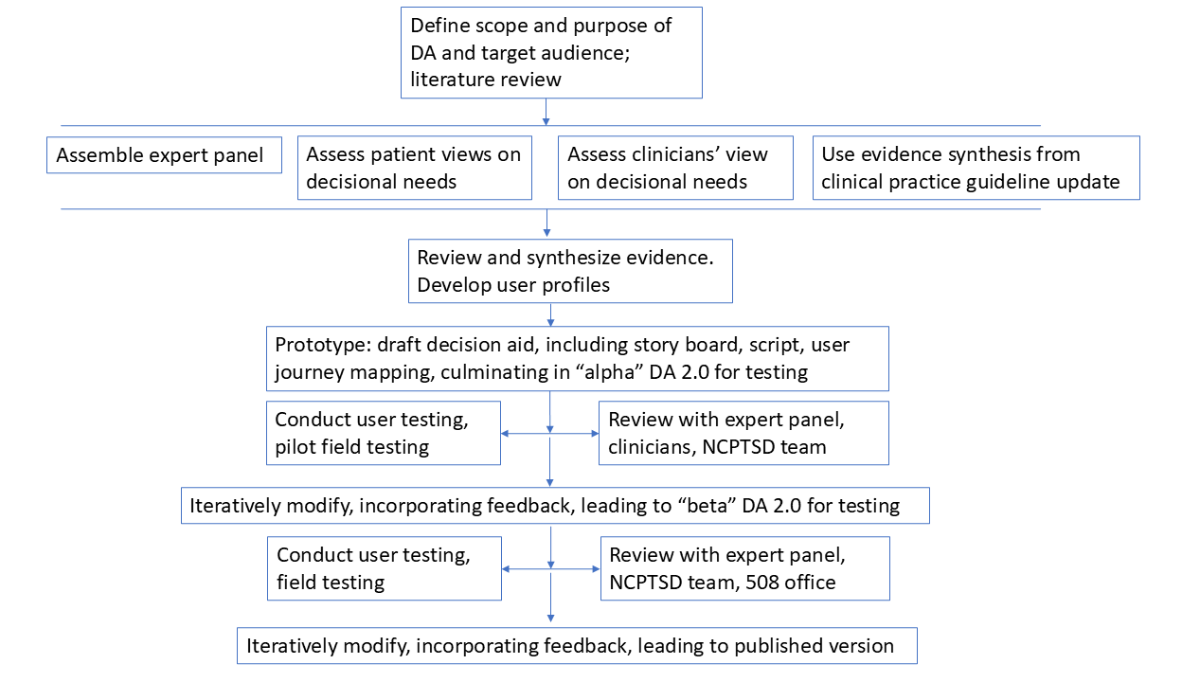
PTSD Treatment DA Development process. DA: decision aid; NCPTSD: National Center for PTSD.

The PTSD DA 2.0 website was developed following the steps outlined in Figure 2.

This process aligns with the one outlined by Coulter et al [10] and was consistent with the development and reporting standards of the IPDAS and Standards for Universal Reporting of Patient Decision Aid Evaluation studies [11], the theory of the Ottawa Decision Support Framework [12], the iterative process outlined by Elwyn et al [13] for web-based DA development, and the principles of user-centered design, in which those who will use a product influence the design at multiple points during the development process [14]. Given that we were working from an existing DA, some background work had already been completed, including assessment of decisional needs, creation of written content, and user experience testing. Although no formal field testing was conducted with the DA 1.0, it has been in use for 8 years by patients and clinicians, thus allowing us to gather informal feedback on how it had been used to date and how it could be more useful in the future.

We used an iterative user-centered process in the development of the DA 2.0, involving multiple stakeholders with the goal of designing a DA that was acceptable to both patients and clinicians in routine clinical practice. There were 3 main iterations of the DA 2.0—the first fully functional draft prototype (“alpha”), followed by the next main iteration (the “beta” version), and finally the public version. Several forms of feedback and testing were used to shape changes between each successive version. The main production groups and stakeholders are outlined below.

#### Project Management Group

The project management group consisted of 2 clinical psychologists (masked) and 1 educational specialist (masked), all NCPTSD members with experience in PTSD, clinical work, research, and health communication. The project management group exercised editorial control and was ultimately responsible for the design of the DA. This group decided on content and design via consensus.

#### Expert Panel

A panel was selected to represent areas of expertise most relevant to the DA development. This involved first identifying areas of expertise and lived experience to be included (PTSD clinician; people who had received PTSD treatment, ie, lived experience, along with experts in SDM in mental health, in DA development, in user experience testing for online products, in health care communication and health care disparities, and in PTSD CPGs), then identifying potential experts in each area, before reaching out to invite them. Attention was also paid to the diversity of demographics as well as experiences. This panel was asked for advice, feedback, and review of design decisions and content through the process of DA development, formally meeting 4 times throughout the process (prior to contract kick-off, after initial prototype development, after alpha development, and after beta development), with additional information-seeking along the way.

#### Technical Production Group

The project management group contracted a company with expertise in web design and user experience testing to produce the website.

#### Veteran Engagement Team

The NCPTSD regularly convenes a panel of veterans to provide feedback on various research and educational products. The DA 1.0 was presented to this team (n=6) prior to the development of the DA 2.0 in order to gain their perspective on needed changes. For instance, they strongly suggested including information for loved ones of people with PTSD, ultimately leading to a dedicated page within the DA with information tailored to loved ones.

#### Other Stakeholders

Care was taken to seek out feedback from experts as well as stakeholders representing each main user type (patients, providers, and loved ones), as recommended for user-centered design of DAs [15]. Before developing the alpha version of DA 2.0, information was sought about what in DA 1.0 worked well and what could be updated or made more user-friendly. This information was sought through individual interviews with a PTSD-focused VA psychiatrist, a VA caregiver support program social worker, the spouse of a veteran with PTSD, and 2 informal focus group-style meetings with VA PTSD-focused psychologists and social workers (n=27). Clinicians comprised a convenience sample of those available locally and nationwide through their involvement in the VA’s PTSD Mentoring and Implementation Program.

Throughout the development process, the education team at the NCPTSD reviewed versions of the website. The team includes clinicians, researchers, communication specialists, and educational specialists who gave feedback on all aspects of design, understandability, representativeness, and utility.

After developing the beta version of the DA 2.0, 7 VA clinicians specializing in PTSD treatment reviewed the content and function of the website, giving their feedback about each (for more patient and provider feedback, see Field Test Descriptive Results section). Finally, the website was submitted for review to ensure that it adhered to Section 508 of the Rehabilitation Act and Section 255 of the Communications Act, ensuring accessibility to those with disabilities as required by US law.

### Sources of Input

The process of updating the DA was conducted in several interrelated and iterative stages, with complementary forms of input at each stage: evidence synthesis, needs assessment, persona development, content and design development (drawing from DA 1.0 content where helpful), user experience testing, and field testing.

#### Evidence Synthesis

The evidence for each intervention included in this DA was largely drawn from the VA/DoD CPG for PTSD (2023) systematic literature review and resulting recommendations. This was supplemented as needed by data from the PTSD Repository, a database of all randomized controlled trials of PTSD treatments that was jointly developed by the NCPTSD and the Pacific Northwest Evidence-Based Practice Center following the Agency for Healthcare Research and Quality’s methods guide for effectiveness and comparative effectiveness reviews [16].

#### Needs Assessment Methods

In preparation for the creation of the DA 2.0, a needs assessment survey was undertaken, similar to one that informed the content and design of the first DA 1.0 [17]. The sample included 887 adults (378 veterans and 509 nonveterans) who screened positive (total score ≥3) for PTSD on the Primary Care PTSD Screen for Diagnostic and Statistical Manual-5 [18]. The sample included women (n=259), men (n=601), and other or did not answer (n=26), with a mean age of 50.75 (SD 16.09) years, largely non-Hispanic White (548/887, 61.8%), along with non-Hispanic Black (117/887, 13.2%), Hispanic (146/887, 16.5%), non-Hispanic 2 or more races (53/887, 6%), or non-Hispanic identifying as “other” (23/887, 2.6%). This survey was supplemented by feedback as outlined in the Other Stakeholders section.

#### Persona Development

At the beginning of the project, user personas were constructed using the expertise of the project management team with input from the expert panel. User journey mapping was used to anticipate the needs of potential users of the site, along with their potential emotional states and decisional needs. This was important given that much of the feedback was from veterans and veteran-facing providers, but the site is designed to be useful for both veterans and civilians.

#### Content and Initial Prototypes

The prior DA 1.0 content was reviewed by the project management team for needed updates, informed by the prior needs assessments. The content was iteratively revised, as the structure of the DA 2.0 was updated. For instance, it was judged that it would not be clear to patients to use the terms “strong recommendation for,” “weak recommendation for,” etc., to describe the varying levels of evidence outlined in the CPG. Rather, this information was consolidated under “effective treatments,” “not enough evidence,” and “not recommended.” The revised content was organized into prototypes, which outline the rough structure of the website along with how users are likely to flow from one page to another. Once prototypes and initial content were finalized, these were reviewed with the expert panel as well.

#### User Experience Testing Methods

First, user experience testing was conducted with 9 individuals who screened positive for PTSD (including a mix of veterans and civilians, sexes, race or ethnicities, ages, socioeconomic backgrounds, geographic US locations, and at least 2 people with low health literacy). In one-on-one moderated sessions, participants were guided through an exploration of the draft website and given specific tasks to complete while being asked to “think out loud” about what they were thinking, noticing, or struggling with. Main tasks across the 2 rounds (9 individuals for review of alpha and 9 for review of beta) included overall site navigation, using the PTSD self-screen, and exploring the “Compare your options” and “Your Summary” pages, along with reviewing veteran-specific and treatment-specific pages. The technical production group and the project management team lead separately summarized impressions of what worked well and what did not in each session, and then discussed to come to a consensus about needed improvements. For example, the alpha testing identified a need to make clearer that this was a government website, and the beta testing identified a need for much clearer feedback from the PTSD self-screen, both of which were implemented in later iterations (for more comprehensive changes, please see the Considerations and Decisions section).

#### Field Testing Methods

Whereas user experience testing allowed a guided exploration of the site with moment-by-moment feedback on specific tasks, field testing was conducted by the project management team to gain the perspective of providers and patients in a real-world setting actually facing the decision at hand. In field testing, VA providers and veterans used the DA as intended in order to test that it was feasible, acceptable, balanced for undecided patients, understood by those with limited reading skills, and delivered with fidelity [11,14]. For this purpose, providers at a VA Medical Center in the Northeastern United States asked patients seeking PTSD treatment if they would like to use and give feedback on the new PTSD DA.

### Measures

#### Overview

For providers who used the DA with their patients, we asked whether the DA would fit into their workflow, whether it was helpful to their patients, and what worked well or could be improved (open-ended).

Patients were asked to complete a brief assessment both before and after using the draft DA 2.0. Measures included basic demographics (without protected health information), a question about how they had used the DA (eg, by themselves or with providers, on a computer or a phone, and for how many minutes), and room for open-ended feedback about what they liked or would improve. Additionally, the following measures were administered (brief measures were prioritized, given that this was part of standard care and not reimbursed research time for providers or patients).

#### Health Literacy Screen

Prior to using the DA, to differentiate those with high from marginal or low health literacy, patients were asked “how confident are you filling out medical forms by yourself?” [19] with a 5-point Likert-type answer from 1=not at all to 5=extremely. Answers of 1=not at all to 3=somewhat suggest low or marginal health literacy, whereas 4=quite a bit to 5=extremely suggest high health literacy.

#### Decisional Self-Efficacy

At both pre- and post-DA, patients completed a decisional self-efficacy scale to measure their belief that they have the ability to make decisions and to participate in SDM [20]. Patients are asked to “show how confident you feel in doing these things by circling the number” with responses on a 5-point Likert-type response scale from 0=not at all confident to 4=very confident. There are 11 items (sample item: “Figure out the choice that best suits me.”).

#### Decisional Conflict (SURE Version)

At both pre- and post-DA, patients completed a decisional conflict scale that measures a state of uncertainty about a course of action [21]. This short version includes 4 items with a dichotomous yes or no response scale. A sample item is: “Do you feel sure about the best choice for you.” Items are summed (with “yes”=1), and a score of 3 or less indicates decisional conflict. The scale was found to have an α of .86 and was responsive to change following use of a DA and counseling [21].

#### Net Promoter Score

Following completion of the DA, satisfaction with the DA was assessed by asking: “How likely is it that you would recommend the PTSD Treatment Decision Aid to other people facing the same decision?” [22]. Responses could range from 0=not at all to 10=extremely likely. Generally, 0 to 6 is considered a “detractor,” 7 or 8 “passive” (ie, satisfied but not happy enough to promote the service), and 9 or 10 a “promoter” (loyal and enthusiastic about the service).

#### Acceptability, Feasibility, and Fidelity

We adapted acceptability questions from O’Connor [23] to assess whether the amount of information in the DA was adequate, information was clear and balanced, and whether this tool would be helpful in making a decision or preparing to talk with a clinician. Assessment of feasibility was set a priori at the ability of 5 or more patients to complete the field testing and give us feedback. Fidelity was operationalized as patients reporting using the DA for at least 15 minutes (ideally with a mix of some viewing on own and some with a clinician, along with some on the phone and some on the computer). Fidelity was further operationalized as providers reporting that the DA would fit into their clinic workflow and that their patients reported they were able to use the DA. We were not able to assess which components of the DA patients used.

### Data Analysis

We analyzed all measures descriptively including frequencies for demographic information, method of using the DA, health literacy, satisfaction, acceptability, feasibility, and fidelity. Provider reports of patient access and fit with workflow were tallied, and patient reports of strengths and weaknesses of the DA were summarized qualitatively. Finally, decisional self-efficacy and decisional conflict were analyzed descriptively to present means and SDs both before and after viewing the DA. Open-ended answers were examined and briefly summarized when they identified either something that worked well or something that could be improved in the DA.

## Results

### Field Test Descriptive Results

In total, 2 patients completed field testing using the alpha DA 2.0, and 6 completed it using the beta DA 2.0. Overall, we received feedback from 4 providers (1 trainee and 3 clinicians, average experience 9.9, SD 17.5 years) and 8 patients (mean age 54.4, SD 17.5 years; men: n=7 and women: n=1; White: n=6 and other race or ethnicity: n=2). This met our feasibility threshold of feedback from at least 5 patients. Of the patients, 6 had likely high health literacy, and 2 had marginal or low health literacy. Most (n=7) completed the DA on a computer (vs n=1 on phone). In total, 4 completed it by themselves, 2 in session with their provider, and 2 both by themselves and in session (amount of time mean 39.38, SD 14.00 minutes; range 20-60 minutes). In terms of acceptability, most (n=7) felt that the amount of information provided was “just right,” that it was clear (n=6), balanced (n=7), and that it was helpful to them in preparing to talk with clinicians (n=8). The average satisfaction was high (mean 8.5, SD 1.9). Suggestions for improvement included enhanced video playback and more veteran testimonials. Participants noted that the strength of the site included the videos, the ability to review options interactively, the ease of saving results, and more than 1 mention of the treatment comparison chart. Of 4 providers, 3 reported that their patients were able to access it (1 reviewed it before asking patients to access); and 3 reported that the DA would fit into their workflow (1 said that, “sort of,” indicating it is helpful in primary care, and that in outpatient care, they would sometimes use it depending on time available).

Given the small sample size, pre- and postchange results are presented descriptively. The decisional conflict and decisional self-efficacy scores were both higher (improved) at posttest (decisional conflict: pre: mean 1.31, SD 1.53; post: mean 3.37, SD 0.74; and decisional self-efficacy: pre: mean 79.83, SD 25.52; post: mean 86.08, SD 18.52).

### Considerations and Decisions

#### Overview

Based on all sources of input and stakeholder feedback outlined earlier, the project management group considered several significant content and design challenges, with decisions on each outlined below. The lead of the project management team (masked) kept a running log of all feedback received from various stakeholders. Feedback was compared and compiled, and the most crucial points were discussed with the expert panel. The project management team made decisions on which pieces to implement with the ultimate goal of improving the user experience in line with DA design principles.

#### Treatments to Include

One decision was which treatments to include in the DA 2.0. This was largely driven by the 2023 CPG alongside a few other considerations. All “recommended” (or first-line) treatments were included in the DA 2.0 (cognitive processing therapy [CPT], prolonged exposure [PE], eye movement desensitization and reprocessing [EMDR], and certain antidepressant medications). We discussed whether and which “suggested” (or second-line) treatments to include, potentially cognitive therapy, present-centered therapy, written exposure therapy, and mindfulness-based stress reduction (MBSR). Additionally, there are numerous other treatments that are either recommended against or considered to have insufficient evidence for inclusion. We considered several factors: (1) the level of availability of treatments (in the United States), (2) the ability for users to have meaningfully different options to select from (eg, having at least 1 nontrauma-focused option), and (3) balancing the desire of our target population for comprehensive information [17] with not overwhelming people with too much information [24]. (4) We also considered that we wanted to help users understand the relatively higher level of evidence for the “recommended” treatments while using language that was not too technical. (5) Finally, IPDAS recommend including information on “doing nothing” as an option. Ultimately, then, we did the following:

- Included information on what to expect if not choosing a treatment (though this was not featured as a highlighted option in our comparison table because there would be almost nothing to say about it). The idea of presenting information about the consequences of doing nothing, without necessarily presenting it as a good option, is reasonable in circumstances where “doing nothing” is not supported by the evidence [25].
- Included information on first-line treatments and helped users understand that all highlighted treatments were evidence-based, but that PE, CPT, and EMDR had the most evidence.
- Included information on “suggested” treatments when they had reasonable availability within the United States and offered a meaningful alternative in some way (eg, for present-centered therapy, offering a nontrauma-focused psychotherapy option; and for written exposure therapy, offering a shorter trauma-focused psychotherapy option with no homework). We did not include cognitive therapy due to limited availability in the United States nor MBSR due to limited availability of the full MBSR option (vs limited mindfulness or meditation options, which are not identified as validated PTSD treatments in the CPG).
- Added a page on treatments not highlighted in the DA called “alternative treatments.” Here, we highlighted other talk therapies, complementary or integrative treatments, medications, biological treatments, and technology-based interventions. We indicated which do not have enough evidence, which are recommended against, and which are effective but not widely available in the United States.
- For each highlighted treatment, offering information about its availability (mainly in the VA, which is where we have the most direct knowledge).

#### Design Format

Some DAs take a fully guided format in which users must start at the beginning and move linearly through to the end. Others are fully flexible, allowing for exploration. We ultimately decided on a flexible format in which we would guide users toward the treatment comparison chart (the “core” of the DA), while also allowing for users to choose their own path. This was felt to be most appropriate partially based on our user profiles and the knowledge that this DA is broadly available (vs only available via a clinician allowing access to it). Therefore, we anticipated that some people would come to this site after a general internet search for information on PTSD treatment and might want more information about PTSD and its treatment in general before choosing a specific treatment. On the other hand, many people use the DA in the context of their clinician, suggesting that they explore it after they have already been diagnosed with PTSD, sometimes having received significant psychoeducation and treatment prior to using this DA. In those cases, they may want to skip directly to information about specific treatments. The flexible format allows clinicians to recommend it to patients at different stages of treatment seeking.

#### Conveying Effectiveness

One challenge is how to best convey the effectiveness of various treatments in a way that is meaningful to patients. Effectiveness is one of the most important pieces of information patients used in making treatment decisions [17]. DAs are expected to include a measure of probable treatment outcome, in a decipherable format, generally a single number compared using the same denominator across all options (eg, comparing 10/100 to 20/100 [26]). This presented a challenge, as there is no clear consensus within the PTSD literature about a standard outcome [27]. Most PTSD treatment trials (and the systematic reviews that aggregate results of these trials) use effect size and tests of statistical significance to determine and communicate treatment effectiveness. These statistics are not easily interpreted by the lay public, nor are they easily converted to a natural frequency (eg, 1 of 10 people). For the DA 1.0, a decision was made to use the loss of a PTSD diagnosis as the metric of change, in part because this has been benchmarked to meaningful change [28], and in part because it was judged to be more interpretable than effect sizes [29]. For the DA 1.0, loss of diagnosis numbers was calculated for each of the treatments included at that time [29], and they were conveyed in the form of icon arrays for ease of interpretation.

This metric, however, was imperfect for a number of reasons. First, loss of diagnosis is not reported in many studies, limiting the data that could be used to calculate these numbers. Second, a number of data points indicated to us that this metric was easily misunderstood by users, especially by veterans. Both veterans and clinicians told us that patients conflated “PTSD” with “trauma,” so they misinterpreted “loss of diagnosis” as meaning that someone would no longer have experienced the trauma or would no longer have symptoms; since this was impossible or unlikely, they interpreted this as an unrealistic and untrustworthy metric. A study found similar results in a qualitative analysis with veterans responding to educational materials with loss of diagnosis statistics [30]. For these reasons, we explored alternatives as we updated for DA 2.0.

We considered an alternative that would use all continuous data from all trials, thereby allowing us to use more of the available data. The challenge, however, was in finding a way to translate this into a dichotomous metric of improvement, which is needed in order to both convey effectiveness simply to the lay public and to be able to compare across treatments. Moreover, it is unclear whether a statistically significant change necessarily leads to meaningful changes in functioning.

Ultimately, we decided to use the same metric (ie, loss of diagnosis) but to label it as “meaningful symptom improvement” instead of “loss of diagnosis.” We reasoned that this would most clearly convey in lay language what was meant by loss of diagnosis. For those who wanted more information, we included a footnote that spells out in more (plain language) detail how we arrived at these numbers, and that they actually involve calculating loss of diagnosis. Data from the PTSD Repository [16] were used to calculate updated effectiveness numbers for each treatment. Specifically, we used randomized controlled trials that were large enough to give reliable estimates (n≥41 per arm), that included one of the highlighted treatments (in individual, outpatient format), and that included a clinician assessment of PTSD diagnosis based on a validated interview. For each treatment arm, we calculated the percentage of participants that no longer met diagnostic criteria for PTSD at posttreatment. Data from inactive “control” arms of the same studies were used to estimate loss of diagnosis numbers for “no treatment.” Standard meta-analysis techniques were then used to combine across individual treatment arms, using properly weighted averages, to arrive at an estimated overall success rate for each treatment type reported.

#### Values and Preferences Assessment

Another criterion for a good DA is that it helps patients identify what they want in a treatment and suggests options that match their preferences. The project management team judged that part of this DA’s goal was to help people identify what they want, ideally with the goal of narrowing down to at most 3 of the 6 possible treatments, to avoid overwhelming patients. The DA 2.0 uses a “decision analysis” [31] approach, in which 4 questions are posed to help people identify their preferences. In total, 2 of the 6 questions from the DA 1.0 were eliminated (group vs individual, since group is not recommended; length of treatment, since that was redundant with medication vs therapy). The research question was modified to help people differentiate between seeing all 6 treatments or only those with the most evidence.

We considered multiple iterations of how those 4 questions would lead to the options presented to users. For instance, it could be that each question is worth one point, and any points that matched a particular treatment were summed to present those with the highest totals. However, using this method, it would be possible for a user to be presented with a treatment that matched many of their preferences but did not match what they said they wanted (eg, indicating not being interested in medications but then having that presented as an option, which may lead to a sense that the algorithm was not working). The questions could also act as a filter (eg, if a user wanted to talk about trauma, the algorithm would filter out those that did not involve a trauma focus), but then, it would be possible to end up with no treatments matching all of a user’s options, which we judged could be discouraging. Ultimately, we decided on a hybrid of those; only a perfect match is highlighted, and if there is not a perfect match, then the wording will make clear that it matches “some” of a user’s preferences. Further, if more than 3 options equally match a user’s preferences, then a flag helps users narrow their options by encouraging them to first consider one of the treatments with the most research support (CPT, PE, and EMDR).

One other consideration was regarding how “neutral” to be in our presentation of information about trauma-focused treatments. A fundamental tenet of DAs is to present information in a fully balanced and objective manner. Yet, there is some debate about whether in some cases it may be most appropriate to deviate from strict neutrality in order to help correct misperceptions [25]. In the case of PTSD treatment, trauma-focused treatments have a stronger evidence base than nontrauma-focused options; yet, a symptom of PTSD is avoidance, which can lead to patients avoiding the most effective treatments. In earlier rounds of feedback, clinicians mentioned that they hoped the DA would encourage patients to consider being willing to talk about (or write about) their trauma. For this reason, after much discussion, we opted to ask patients whether they were open to talking about their trauma, but we also presented basic information about why it might be useful as well as a link to a quote from a veteran about his experience with talking about trauma in PTSD treatment. The full text of the question thus reads:

You may have different responses to the idea of talking, writing, or thinking about your trauma. In your day-to-day life when you are reminded about your trauma, you may get upset or overwhelmed. Talking with a clinician can be different. Talking about the trauma can help you gain more control of your thoughts and feelings. It can be hard at first. But many people find that, over time, it helps them feel better. How open are you to talking or writing about your trauma?

### Other Changes Made in Response to Feedback

Taking into account all forms of feedback, we made iterative changes at each stage of the development process as part of the user-centered design process [15]. For instance, in response to early needs assessment results, we made extensive wording changes throughout the website to make it more accessible and understandable to the target audience. This included changing from “your plan” (which sounded more clinical) to “your summary” (indicating a summary of all answers entered into the DA), “psychotherapy” to “talk therapy” (based on feedback from multiple stakeholders), and “loss of diagnosis” to “meaningful symptom improvement” (as outlined earlier). In response to user experience testing, we made several changes to enhance user experience, such as more clearly indicating—through the use of visual cues—which treatments matched a user’s preferences, adding a within-page navigation menu on the treatment detail pages, and breaking up long paragraphs of information into more digestible smaller chunks or bullet points. In response to field testing with veterans with low health literacy, we added the ability to control the playback speed of the videos and added information about low health literacy to the clinician’s page. In response to user experience testing, we made it easier to find information about how to find a clinician if they do not have one. We also simplified language and made clearer that the PTSD self-screen is a screen only and that a clinician is needed for a formal diagnosis. In response to the expert panel, we clarified that completing this DA is only one part of an SDM process, and that an important next step is a discussion with a clinician. In several other areas, we tried to clarify and simplify language, such as by clarifying which studies were used to identify treatment efficacy and standardizing our descriptions of alternative treatments and their potential side effects.

### Quality Criteria

The IPDAS Collaboration updated their checklist of quality metrics for DAs [7]. The updated version was released in March 2025, when we were most of the way through creating the DA 2.0 (see Multimedia Appendix 1 for full checklist). We compared the DA 2.0 to the criteria on the checklist and made a few final edits in order to meet the new criteria. There are 7 criteria that are considered qualifying (must meet to be defined as a DA), 7 considered essential (to reduce risk of harmful bias), and 46 considered enhancing (desirable but not essential). Of these, we met all 14 of the qualifying and essential criteria, along with 41 of 46 enhancing criteria (the other 5 were rated N/A). We specifically checked with the 2 DA experts (YZ-I and KBE) on our expert panel to ensure that we were adequately addressing the criteria. We also followed the principles of user-centered design for personal health tools as indicated by the 11-item checklist in Multimedia Appendix 2 [14].

## Discussion

This paper describes the systematic process of developing an updated PTSD Treatment DA 2.0. The DA 2.0 includes information on PTSD, treatment, and the risks and benefits of each major treatment option. The final version was identified as balanced, fair, and acceptable in the small sample of pilot field test patients and providers. It also met all original and updated criteria of the IPDAS Collaboration.

The plan for dissemination builds on existing dissemination workflows. Given that this DA is designed to be completed either on one’s own or in collaboration with a provider (though ultimately leading to a discussion with a provider), it is freely available on the internet [9]. This means anyone, at any time, can access the tool. The hope is that the printable “Your Summary” page will allow users to save their treatment preferences and share them with a provider—even if that provider was unaware of the DA. Additionally, the DA is promoted to providers and patients via the NCPTSD website, promotional videos on YouTube, social media postings by the NCPTSD’s social media presence, a VA news article, emails to providers, internal VA presentations, and various promotional materials available to providers (eg, a flyer, wallet card, and “prescription pad”).

Some limitations and future directions should be noted. This paper only reports on initial field testing. To date, the current DA has not been tested in a larger randomized controlled trial, which would be beneficial to more rigorously test its benefits and drawbacks. For instance, fidelity was assessed in a limited way, in that, we were not able to assess which parts of the DA were viewed. It would be helpful to identify whether the DA leads to more informed and values-congruent decisions, less distress, and better treatment engagement and outcomes. Additionally, it would be valuable to examine the DA as part of a larger SDM intervention, given that there is to date relatively less research on SDM than on DAs. In doing so, it may be helpful to specifically sample for users with low health literacy.

In conclusion, the newly developed PTSD Treatment DA 2.0 aims to support patients, providers, and loved ones in learning more about guideline-recommended treatments. Although significant evidence now exists to support the utility of DAs generally, remarkably little research has so far focused on either DAs or SDM in the area of PTSD. We hope that this tool will be of help to those seeking to recover from this treatable illness.

## Appendix

Multimedia Appendix 1 International Patient Decision Aid Standards guidelines.

Multimedia Appendix 2 User- and human-centered design for personal health tools.

## Abbreviations

CPG: Clinical Practice Guideline
CPT: cognitive processing therapy
DA: decision aid
EMDR: eye movement desensitization and reprocessing
HIPAA: Health Insurance Portability and Accountability Act
IPDAS: International Patient Decision Aid Standards
IRB: Institutional Review Board
MBSR: mindfulness-based stress reduction
NCPTSD: National Center for PTSD
PE: prolonged exposure
PTSD: posttraumatic stress disorder
SDM: shared decision-making
VA: Veterans Administration
VA/DoD: Veterans Affairs/Department of Defense
