# Correction: Posttraumatic Stress Disorder Treatment Decision Aid: User-Centered Design Update Approach

**DOI:** 10.2196/106657

**Published:** 2026-08-14

**Authors:** Sadie E Larsen, Cybele Merrick, Yaara Zisman-Ilani, Karen B Eden, Shanna Treworgy, Jessica L Hamblen

**Affiliations:** 1 National Center for Posttraumatic Stress Disorder White River Junction, VT United States; 2 Department of Psychiatry and Behavioral Medicine Medical College of Wisconsin Milwaukee, WI United States; 3 Temple University Philadelphia, PA United States; 4 University College London London, England United Kingdom; 5 Oregon Health & Science University Portland, OR United States; 6 White River Junction VA Medical Center White River Junction, VT United States; 7 Geisel School of Medicine at Dartmouth Hanover, NH United States

In “Posttraumatic Stress Disorder Treatment Decision Aid: User-Centered Design Update Approach” [1], the authors made one correction.

The following sentence from the Results section was revised:

Specifically, we used randomized controlled trials that were large enough to give reliable estimates (n≥41), that included one of the highlighted treatments...

The sentence now reads:

Specifically, we used randomized controlled trials that were large enough to give reliable estimates (n≥41 per arm), that included one of the highlighted treatments...

The correction will appear in the online version of the paper on the JMIR Publications website, together with the publication of this correction notice. Because this was made after submission to PubMed, PubMed Central, and other full-text repositories, the corrected article has also been resubmitted to those repositories.
