# Supplementary material for: Posttraumatic Stress Disorder Treatment Decision Aid: User-Centered Design Update Approach
Source: JMIR Form Res. 2026 Jun 10;10:e89074. doi: 10.2196/89074 (PMC13294652; doi:10.2196/89074)
Supplement: Multimedia Appendix 1 [file formative_v10i1e89074_app1.docx]

| **IPDAS criteria** | **How the criteria were addressed (when relevant)** | **Location within the PTSD Treatment Decision Aid** |
| --- | --- | --- |
| **Qualifying Criteria (mandatory)** | | |
| Describes the health condition |  | Landing page: “What is PTSD? Posttraumatic stress disorder (PTSD) is a mental health problem that can occur after someone goes through a traumatic event like combat, assault, or a disaster. Most people have some stress reactions after a trauma. Going through a trauma does not mean you'll get PTSD. But if the reactions disrupt your life and don't go away over time, you may have PTSD.  There are four main types of PTSD symptoms:   - Reliving the event - Avoiding reminders of the event - Negative changes in beliefs and feelings - Feeling on edge or keyed up”   There are links to learn more about PTSD on the “Understanding PTSD” page. |
| Explicitly states the decision to be considered |  | Landing page: “The PTSD Treatment Decision Aid will help you learn about effective treatment options for posttraumatic stress disorder (PTSD). It will give you the facts you need to make an informed decision.  On this website, you can: …   - Build a chart to compare the treatments you think might work best for you.” |
| Identifies the target audience |  | About this tool and your privacy page (bottom of landing page): “Target audience  The PTSD Treatment Decision Aid was developed for use by people who have been diagnosed with PTSD, those who suspect they may have PTSD, and family and friends of loved ones with PTSD symptoms.” |
| Lists options including if relevant, “wait and see” (e.g., making no change, doing nothing) |  | All main treatment options listed on “Compare your options” page. On “Explore Treatments” page, there is also information on no treatment: “**What if I have PTSD but do not get treatment?**  Sometimes people with PTSD assume their PTSD symptoms will go away with enough time. This might be the case for some people, but not for everyone.  Most people who are going to get better without treatment get better in the first year after a trauma. So, if you have had your symptoms for more than a year, they are likely to continue without treatment.” |
| Describes positive features of options (benefits) |  | The comparison chart on “Compare your options” page allows for comparison of benefits (mainly “Is it effective?”) |
| Describes negative features of options (harms) |  | The comparison chart on “Compare your options” page allows for comparison of harms (mainly “What are the risks?”) |
| Asks patients to think about which positive and negative features of options matter most to them OR describes what it is like to experience the consequences of options (physical, psychological, social) |  | The “Compare your options” page includes four preference questions to help people evaluate the different options. |
| **Essential Criteria (must have to reduce risk of harmful bias)** | | |
| Is based on best available evidence that is, where possible, directly applicable to the patients and clinicians using it. | The treatment options are based on the VA/DoD PTSD Clinical Practice Guidelines. These recommendations are largely consistent with other leading clinical practice guidelines (e.g. American Psychological Association). | The “Explore treatments” page indicates this: “Treatments in the PTSD Treatment Decision Aid are recommended in the 2023 guideline published by the Department of Veterans Affairs and Department of Defense (VA/DoD).” |
| Describes how potential users were involved in steps of designing, developing and/or refining a prototype (new) |  | On the “About this tool and your privacy page”: “People with PTSD were involved in creating the PTSD Treatment Decision Aid in several ways. First, we asked a panel of Veterans—some with PTSD—for their opinions on how to update the prior decision aid. Second, we included Veterans with PTSD on an expert panel that advised on multiple revisions of this decision aid. Third, we asked both Veterans and civilians with PTSD to test versions of the decision aid as we updated it. Finally, clinicians and patients at one Veterans Affairs hospital used versions of the decision aid and gave us feedback. Each of these steps provided feedback that shaped the current PTSD Treatment Decision Aid.” |
| Shows negative/positive features of options in a balanced manner (e.g., neutral, unbiased, non-directive, complete) | In field testing, we asked patients “Did the information about different treatment options seem   - Balanced - Slanted toward some options___”   All patients indicated that they found it balanced. | Both positive and negative features are compared side-by-side on the “Compare your options” page. |
| Reports where the money came from to develop the PDA and it is clearly stated (e.g., plain language, prominent) (revised) |  | On the “About this tool and your privacy page”: “This website was funded by the Veterans Affairs National Center for PTSD.” |
| Provides complete citations to evidence selected |  | On each individual treatment page, a footnote has a link to the list of all studies (labeled “Footnotes and list of studies”). |
| Provides a production or publication date |  | On the “About this tool and your privacy page”: “The initial version of the PTSD Treatment Decision Aid was released in April 2017 and updated in June 2017 to reflect changes to the 2017 PTSD Clinical Practice Guideline published by the Department of Veterans Affairs and Department of Defense. A substantially updated version was released in August 2025 to reflect the updated 2023 VA/DoD PTSD Clinical Practice Guideline.” |
| Provides information about the proposed update policy (or available supporting document) (same) |  | On the “About this tool and your privacy page”: “Major updates will be conducted when future VA/DoD Clinical Practice Guidelines are released.” |
| **Enhancing Criteria (desirable but not essential)** | | |
| Makes it possible to compare benefits and harms for features of available options side-by-side |  | The “Compare your options” page features this side-by-side comparison of benefits and harms. |
| Describes the natural course of the health condition if no healthcare option is chosen |  | The “Explore treatments” page includes this information: “**What if I have PTSD but do not get treatment?**  Sometimes people with PTSD assume their PTSD symptoms will go away with enough time. This might be the case for some people, but not for everyone.  Most people who are going to get better without treatment get better in the first year after a trauma. So, if you have had your symptoms for more than a year, they are likely to continue without treatment.” |
| Presents essential content with guidance on how and where patients can seek additional information to support decision making |  | On the “About this tool and your privacy page”: “How did we decide which treatments to include in the PTSD Treatment Decision Aid? Treatments in the PTSD Treatment Decision Aid are recommended in the 2023 PTSD Clinical Practice Guideline published by the Department of Veterans Affairs and Department of Defense (VA/DoD). The workgroup for that guideline reviewed all relevant research using the [GRADE](https://www.gradeworkinggroup.org/) approach, a rigorous methodology for assessing the strength of the treatment evidence. The workgroup found that the positive effects of these treatments outweigh the risks. For more information see the [clinical practice guideline](https://www.healthquality.va.gov/guidelines/MH/ptsd/VA-DOD-CPG-PTSD-Patient-Summary.pdf).” |
| Presents information about outcomes of options (positive and negative) including the chances they [may] happen, if reliable estimates are available |  | On “Compare your options” page, there is information about positive and negative outcomes, including chances of meaningful symptom reduction, for which we have reliable estimates. |
| Presents probabilities using both positive and negative frames (e.g., showing both survival and death rates) | N/A |  |
| Presents probabilities using numbers rather than words in general. Care should be taken if numbers and words are combined |  | Probabilities of meaningful symptom improvement are presented using numbers on the “Compare your options” page and on each individual treatment page. |
| Presents probabilities using event rates in a defined group of patients for a specified time |  | Probabilities of meaningful symptom improvement are presented for patients who receive a given treatment over a time frame of “about 3 months”. |
| Compares probabilities of options using common denominator formats (e.g., probabilities or common denominator (frequencies)) |  | Probabilities of meaningful symptom improvement are presented as “out of every 100 people” for every option. |
| Uses the same scales in the diagrams comparing options |  | On each individual treatment page, the chances of improvement are compared between 100 people who receive a treatment versus 100 people who do not receive treatment. |
| Describes the uncertainty around the probabilities (e.g., by giving a range or by using phrases such as 'our best guess is') (changed from essential to enhancing) | We opted not to include this information for the sake of clarity as we were not able to identify an appropriately clear and user-friendly way to convey this. |  |
| Uses the same time frame for all options and outcomes, if time-based risk formats are used |  | Probabilities of meaningful symptom improvement are all presented “after about 3 months”. |
| Uses visual displays (e.g., icon arrays, stacked bar graphs) that show both the numerator and the denominator (i.e., the part-to-whole relationship) |  | Probabilities of meaningful symptom improvement are all presented in icon arrays showing the numerator and denominator on the individual treatment pages. |
| Places the chances of what might happen in the context of other situations (e.g., chances of developing other diseases, dying of other diseases, dying from any cause) | We opted not to do this as there was no clear and very relevant comparison. |  |
| Uses risk formats that were tested with end users in the population to whom the risk applies | All effectiveness numbers were utilized in user testing and field testing, with no difficulties in understanding them. |  |
| Uses an explicit values clarification method to help patients clarify what it is important to them in deciding upon options |  | The “Compare your options” page includes 4 preference questions to help identify what people want in a treatment. |
| Provides a step-by-step way to make a decision |  | The homepage outlines a step-by-step process: “On this website, you can:   - Read about the treatments or watch videos explaining how they work. - Build a chart to compare the treatments you think might work best for you. - Get ideas for how to talk with your medical team about your options.”   The website is designed to be guided but to also allow free-form exploration. |
| Includes tools like worksheets or lists of questions to use when discussing options with a health professional |  | “Your summary” is a prominent page that includes information entered into the decision aid and prompts to include treatment goals and questions. |
| Indicates which section of the PDA where each citation was used |  | Where the VA/DoD Clinical Practice Guidelines were used to select treatments, that is indicated (on “Explore treatments”). Other specific articles were used to calculate numbers of symptom improvement – those are linked from the bottom of each specific treatment page. |
| Reports the source of the personalized evidence, if risk estimates or risk management options are personalized to individual characteristics | N/A |  |
| Describes how research evidence was searched for, appraised, selected, and synthesized (derived from systematic reviews or evidence-based clinical practice guidelines, where possible) |  | On each individual treatment page, a footnote reads: “To see how helpful treatments are, the PTSD Treatment Decision Aid shows how many people improved after getting a treatment. If people improve enough that their symptoms no longer meet criteria for a diagnosis of PTSD, this is called “loss of diagnosis.” To estimate how many people would have a loss of diagnosis after each treatment, we gathered data from studies of the treatments included in the PTSD Treatment Decision Aid. We only included studies that were large, compared the treatment to another treatment, and used clinicians to assess whether patients had PTSD.” |
| Describes the quality of the research evidence used (e.g., using the GRADE approach) |  | On the “About this tool and your privacy page”:  “How did we decide which treatments to include in the PTSD Treatment Decision Aid?  Treatments in the PTSD Treatment Decision Aid are recommended in the 2023 PTSD Clinical Practice Guideline published by the Department of Veterans Affairs and Department of Defense (VA/DoD). The workgroup for that guideline reviewed all relevant research using the [GRADE](https://www.gradeworkinggroup.org/) approach, a rigorous methodology for assessing the strength of the treatment evidence. The workgroup found that the positive effects of these treatments outweigh the risks. For more information see the [clinical practice guideline](https://www.healthquality.va.gov/guidelines/MH/ptsd/VA-DOD-CPG-PTSD-Patient-Summary.pdf).” |
| Designed, formatted and written at a level to be understood by its target audience including people with lower health literacy. | A range of health literacy was considered from the beginning. For user testing, we specifically sampled for multiple people with low health literacy. Plain language was used throughout. | On “About this tool and privacy”: “The decision aid, except for content specific to clinicians, is written in plain language, with an overall Flesch-Kincaid readability score of 7.7.” |
| Uses strategies to reduce cognitive burden (e.g., plain language; glossary of key terms; bullet points; simple navigation), by providing non-text ways to help patients understand information (e.g., visual cues and illustrations, audio narration, video) | Throughout the decision aid, plain language was prioritized. Large batches of text were broken up using bullet points. There are multiple videos throughout the decision aid to present information in different formats. |  |
| Uses field testing to show that the PDA was understood by patients with lower health literacy | We conducted field testing with eight individuals, two of whom had low health literacy. Field testing suggested overall lower ratings of the DA by those with low health literacy, however, they did report increased decisional self-efficacy and less decisional conflict after viewing the DA. |  |
| Developed in accordance with health literacy guidelines, for example by meeting recommended thresholds of the Patient Education Materials Assessment Tool (PEMAT; >70%) and a grade reading level of 8 or lower | A range of health literacy was considered from the beginning. For user testing, we specifically sampled for multiple people with low health literacy. Plain language was used throughout. | On “About this tool and privacy”: “The decision aid, except for content specific to clinicians, is written in plain language, with an overall Flesch-Kincaid readability score of 7.7.” |
| Reports how co-design was used in its development | N/A |  |
| Reports that the PDA was developed without using money from a source that stands to gain or lose by the choices patients make |  | On “About this tool and your privacy”: “The developers of the PTSD Treatment Decision Aid and their affiliated institutions do not stand to gain or lose anything based on the choices people make after using the decision aid.” |
| Reports that no authors stand to gain or lose by the choices patients make after using the PDA |  | On “About this tool and your privacy”: “The developers of the PTSD Treatment Decision Aid and their affiliated institutions do not stand to gain or lose anything based on the choices people make after using the decision aid.” |
| Reports that no authors’ affiliations stand to gain or lose by the choices patients make after using the PDA |  | On “About this tool and your privacy”: “The developers of the PTSD Treatment Decision Aid and their affiliated institutions do not stand to gain or lose anything based on the choices people make after using the decision aid.” |
| Includes authors'/developers' credentials or qualifications |  | On “About this tool and your privacy”: “The content was developed by a team of clinical psychologists and education specialists at the National Center for PTSD with expertise in PTSD treatment, mental health, and shared decision-making.” |
| Reports where the money came from to copy and distribute the PDA and it is clearly stated (e.g., prominent, written in plain language) | N/A |  |
| Includes information about the expertise of the authors/developers (e.g., patients/caregivers, patient advocates, nurses, physicians) |  | On “About this tool and your privacy”: “The content was developed by a team of clinical psychologists and education specialists at the National Center for PTSD with expertise in PTSD treatment, mental health, and shared decision-making.” |
| Reports that potential users (e.g., patients, health care professionals, caregivers) were involved in steps to help understand user goals, motivations, needs, and expectations specific to the decision |  | On “About this tool and your privacy”:  “Were clinicians and people with PTSD involved in creating the PTSD Treatment Decision Aid?  Yes. The PTSD Treatment Decision Aid has gone through several revisions, with each revision led by psychologists and educational specialists at the National Center for PTSD. Input has been gathered from two national surveys of Veterans and civilians with PTSD, along with interviews with patients, Veterans, and clinicians. We would also like to thank many clinicians and Veterans for contributing to the videos in this decision aid.  People with PTSD were involved in creating the PTSD Treatment Decision Aid in several ways. First, we asked a panel of Veterans—some with PTSD—for their opinions on how to update the prior decision aid. Second, we included Veterans with PTSD on an expert panel that advised on multiple revisions of this decision aid. Third, we conducted usability testing on each major version of the decision aid with both Veterans and civilians with PTSD. Finally, clinicians and patients at one Veterans Affairs hospital used versions of the decision aid and gave us feedback. Each of these steps provided feedback that shaped the current PTSD Treatment Decision Aid. ” |
| Involved potential users in steps intended to evaluate prototypes of the PDA |  | On “About this tool and your privacy”:  “Were clinicians and people with PTSD involved in creating the PTSD Treatment Decision Aid?  Yes. …  People with PTSD were involved in creating the PTSD Treatment Decision Aid in several ways. First, we asked a panel of Veterans—some with PTSD—for their opinions on how to update the prior decision aid. Second, we included Veterans with PTSD on an expert panel that advised on multiple revisions of this decision aid. Third, we conducted usability testing on each major version of the decision aid with both Veterans and civilians with PTSD. Finally, clinicians and patients at one Veterans Affairs hospital used versions of the decision aid and gave us feedback. Each of these steps provided feedback that shaped the current PTSD Treatment Decision Aid. ” |
| Describes how evaluation showed that undecided patients found the information was presented in a balanced way | Please see main manuscript. Of eight field test patients, seven reported the information was balanced, and one reported that it was both balanced and slanted (without further clarification given). |  |
| Describes how evaluation showed that it was acceptable to potential users | Please see main manuscript. Of eight field test participants, seven indicated that the amount of information was “just right”, with one indicating that it was “Too little for the average person, but much more helpful to review with a provider”. Six found the information clear, two indicated it was some of both clear and unclear (without further clarification given). All agreed that it was helpful to prepare them for making a decision or talking with a provider about treatment options. |  |
| Reports that potential users were observed using the PDA | This is reported in the associated manuscript (see “User Testing” section). |  |
| Uses iterative cycles of feedback from potential users of the PDA (e.g., patients/public, healthcare professionals) in the development | Please see main manuscript. Indeed, multiple iterations of the decision aid were tested, each of them with feedback from potential users of the decision aid (both patients and providers, both user testing and field testing). |  |
| Reports explicit changes between iterative cycles | Please see main manuscript for a list of changes (in the section “Changes Made in Response to Feedback”). |  |
| Includes relevant experts on the development team (e.g., potential users, clinical content/subject matter experts, patients/members of the public who have faced the decision or could reasonably be expected to face the decision in the future, experts in plain language, accessibility, design, engineering, digital security, decision scientists, biostatisticians, epidemiologists, implementation scientists) | This information is included in “About this tool and your privacy”. In short, the content was developed by a team at the National Center for PTSD with expertise in PTSD treatment, mental health, and shared decision-making. An expert panel advised on each stage of development and consisted of patients, clinical psychologists, clinical social workers, researchers, and experts in shared decision-making, decision aids, health communication, healthcare disparities, and clinical practice guidelines. Field testing also involved actual clinicians and patients facing the decision of which PTSD treatment to choose. The contractor who built the site had further expertise in plain language, accessibility, design, and adult learning principles. |  |
| Reports that members of equity-deserving populations were meaningfully involved in development of PDA, when relevant | Attention was paid to inclusion of diverse members on the expert panel and in user testing. |  |
| Describes how the PDA was culturally adapted from existing PDAs, where appropriate | N/A |  |
| Follows a theoretical framework or conceptual model together with IPDAS criteria for development | IPDAS criteria were closely attended to throughout as documented here. The conflict model of decision-making guided the DA development and assessment (e.g., O'Connor AM. Validation of a decisional conflict scale. *Medical Decision Making* 1995;15:25-30.; Elwyn, G., Stiel, M., Durand, M. A., & Boivin, J. (2011). The design of patient decision support interventions: addressing the theory–practice gap. *Journal of evaluation in clinical practice*, *17*(4), 565-574.). |  |
| There is evidence that the PDA helps patients: - recognize that a decision needs to be made  - know about the available options  - know about different features of options  - understand that values affect the decision  - be clear about which features of options matter most to them  - discuss values with their health professionals - become involved in decision making in ways they prefer  - improves the match between the features that matter most to the informed patient and the option that is chosen | The decision aid was tested in two ways: via usability testing (on the “alpha” and “beta” versions), and field testing. Both forms of testing indicated that people could identify the available treatments and features of those treatments. More in-depth field testing would be needed to address the remaining questions. |  |
| If any evaluation of the PDA was conducted, reports the findings with attention to SUNDAE guidelines (Standards for UNiversal reporting of patient Decision Aid Evaluation) | Please see main manuscript which conforms to the SUNDAE guidelines for reporting on development of a decision aid. |  |
| Describes how evidence of PDA effectiveness was gathered using instruments that have strong psychometric properties (i.e., the evaluation tool is valid and reliable) | Please see main manuscript. All instruments used in the field test were validated measures and/or recommended as part of the Ottawa evaluation process. |  |
