# Supplementary material for: Posttraumatic Stress Disorder Treatment Decision Aid: User-Centered Design Update Approach
Source: JMIR Form Res. 2026 Jun 10;10:e89074. doi: 10.2196/89074 (PMC13294652; doi:10.2196/89074)
Supplement: Multimedia Appendix 2 [file formative_v10i1e89074_app2.docx]

| **User-Centered Design Criteria** | **Yes/No** | **How the Criteria were Addressed** |
| --- | --- | --- |
| Were potential end users (eg, patients, caregivers, family and friends, surrogates) involved in any steps to help understand users (eg, who they are, in what context might they use the tool) and their needs? | Yes | Yes, people with PTSD and loved ones were consulted prior to developing the alpha prototype (via a needs assessment survey with both Veterans and civilians with PTSD symptoms, and a focus group with Veterans, some with PTSD). |
| Were potential end users involved in any steps of designing, developing, and/or refining a prototype? | Yes | The expert panel included two people who had received PTSD treatment. They reviewed and advised on initial design and prototype refining. |
| Were potential end users involved in any steps intended to evaluate prototypes or a final version of the tool? | Yes | Between alpha, beta, and published version of the DA, there was user experience testing and field testing of each major iteration that included potential end users (i.e., people with PTSD symptoms). |
| Were potential end users asked their opinions of the tool in any way? | Yes | In both user experience testing and field testing, end users were asked their opinion of the tool (not only with closed-ended questions, but also with open-ended questions). |
| Were potential end users observed using the tool in any way? | Yes | User experience testing involved observing end users (people with PTSD symptoms) using the tool, in both the alpha and beta versions. |
| Did the development process have 3 or more iterative cycles? | Yes | Initial wireframes and then two main prototypes were developed prior to the final published DA; before and after each prototype there were several rounds of iterative revision with review by people outside the project management team. |
| Were changes between iterative cycles explicitly reported in any way? | Yes | Main revisions are reported in the associated manuscript. A more detailed list of revisions was kept as part of the internal technical report process. |
| Were health professionals asked their opinion of the tool at any point? | Yes | Health professionals were consulted both before the development of the new DA and after each major iteration. |
| Were health professionals consulted before the first prototype was developed? | Yes | Health professionals were consulted in two focus groups prior to the first prototype. |
| Were health professionals consulted between initial and final prototypes? | Yes | Health professionals gave feedback on the alpha DA. Health professionals also gave feedback as part of the field testing that occurred with both the alpha and the beta DAs. |
| Was an expert panel involved? | Yes | An expert panel was composed of experts in shared decision-making in mental health, in DA development, in user experience testing for online products, in healthcare communication and healthcare disparities, and in PTSD clinical practice guidelines. In addition, one PTSD clinician and two lived experience experts (with PTSD) were included in the panel. The expert panel was convened four times: before prototype development, after initial wireframes, and after each major iteration. |
